# The cut-off value of tumor size and appropriate timing of follow-up for management of minimal EUS-suspected gastric gastrointestinal stromal tumors

**DOI:** 10.1186/s12876-016-0567-4

**Published:** 2017-01-11

**Authors:** Zhidong Gao, Chao Wang, Qian Xue, Jingtong Wang, Zhanlong Shen, Kewei Jiang, Kai Shen, Bin Liang, Xiaodong Yang, Qiwei Xie, Shan Wang, Yingjiang Ye

**Affiliations:** 1 Department of Gastrointestinal Surgery, Peking University People’s Hospital, Beijing, 100044 People’s Republic of China; 2 Department of Gastroenterology and Endoscopy, Peking University People’s Hospital, Beijing, 100044 People’s Republic of China

**Keywords:** Endoscopic ultrasound (EUS), Stomach, Gastrointestinal stromal tumor (GIST)

## Abstract

**Backgroud:**

The detectable rate of minimal gastric GISTs has continuously increased. While the surveillance and management of GIST <2 cm have been deemed controversial or lack evidence-based approaches.  The aim of the current study is to propose a cut-off value of tumor size for treatment policy and the appropriate timing for endoscopic ultrasonography (EUS) follow-up in the minimal EUS-suspected gastric GIST patients.

**Methods:**

A single-institution retrospective study was performed. 69 patients with EUS-suspected gastric GISTs were studied from November 2008 to March 2015. 69 patients with minimal gastric GISTs ≤2 cm diagnosed by EUS were followed for a mean period of 29 months (range, 12 to 70). An at least 20% increase of the maximal diameter of the tumors was set as a significant change.

**Results:**

During follow-up, Of the 69 minimal EUS-suspected GISTs, 16 (23.2%) showed significant changes in size. 11 out of 69 GISTs (15.9%), 6 out of 43 GISTs (14.0%), 7 out of 30 GISTs (23.3%) showed significant changes in size, at 1 year, 2 years, and more than 3 years respectively. The receiver operating characteristic curve analysis showed that the tumor size cut-off was 9.5 mm. Only 4.7 and 3.7% of gastric EUS-suspected GISTs of <9.5 mm in size showed significant changes at 1 year and 2 years, while 9.5% at more than 3 years. 34.6, 31.3 and 55.6% of gastric EUS-suspected GISTs of ≥ 9.5 mm in size showed significant changes at 1 year, 2 years and more than 3 years.

**Conclusions:**

Minimal EUS-suspected GISTs, larger than 9.5 mm may be associated with significant progression. The patients with a ≥ 9.5 mm GIST should have a EUS 6–12months, while <9.5 mm GIST may have a EUS extended to every 2–3 years.

**Electronic supplementary material:**

The online version of this article (doi:10.1186/s12876-016-0567-4) contains supplementary material, which is available to authorized users.

## Background

Gastrointestinal stromal tumors (GISTs) are the most common primary mesenchymal tumors in the gastrointestinal tract and span a clinical spectrum from benign to malignant. GISTs occur anywhere along the gastrointestinal tract, but they are most common in the stomach (50–60%) [1]. Owing to the popularization of EUS, the detectable rate of gastric GISTs has continuously increased, specifically for minimal gastric GISTs (diameter less than 2 cm). Surgery is the treatment for primary, local gastric GISTs larger than 2 cm, while conservative follow-up is suggested for lesions less than 2 cm. In fact, the National Comprehensive Cancer Network (NCCN) and the European Society of Medical Oncology (ESMO) guidelines for the surveillance and management of GIST <2 cm have been deemed controversial or lack evidence-based approaches. Endoscopic ultrasonography (EUS) has been utilized to diagnose gastric GISTs with high accuracy, sensibility, and specificity (87, 95, 72%, respectively) [2, 3]. The typical EUS finding of a gastric GIST is a hypoechoic lesion arising from the fourth layer of the gastric wall. The goal of this study was to evaluate the malignant potential of minimal gastric GISTs to determine the best cut-off value for tumor size and appropriate timing for EUS follow-up, in order to provide clinical evidence for malignant potential in the management of minimal gastric GISTs.

## Methods

### Study design and population

This retrospective study was approved by our institutional ethics committee and meets the guidelines of our responsible governmental agency. It reviewed data from patients with minimal gastric GISTs diagnosed by using EUS at Peking University People’s Hospital between November 2008 and March 2015.

Tumor size was determined with the maximum diameter obtained by using EUS. The criterion of minimal gastric GISTs diagnosed by using EUS is a hypoechoic lesion arising from the fourth layer of the gastric wall. Every patient had signed a consent form before EUS inspection. All EUS image files were reviewed by a single experienced EUS endoscopist (JW).

**Fig. 1:**
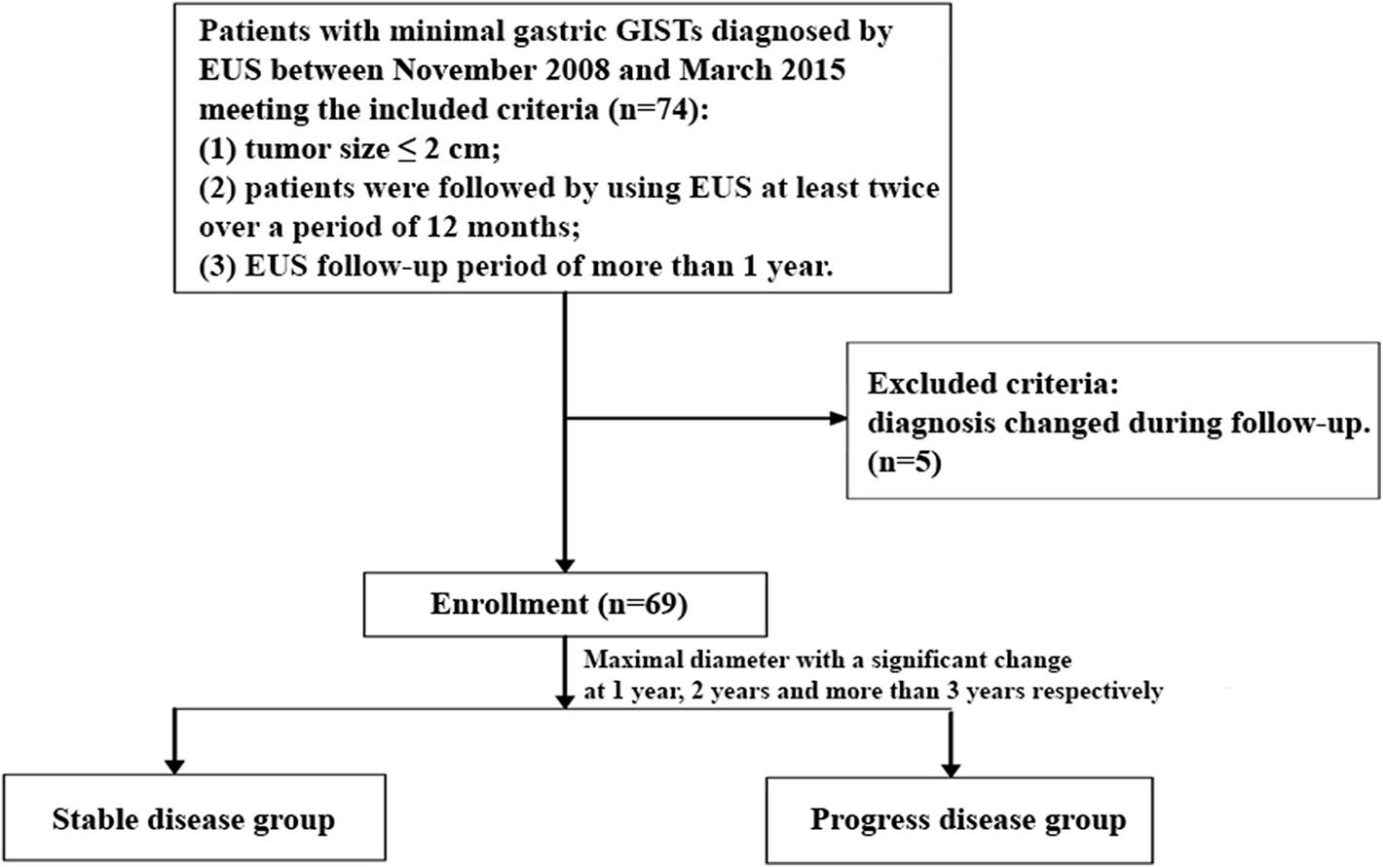
Flow diagram of enrollment patients

Inclusion criteria for this study were: (1) tumor size ≤ 2 cm; (2) patients were followed by using EUS at least twice over a period of 12 months; (3) EUS follow-up period of more than 1 year. The exclusion criteria were: (1) cancer patients; (2) diagnosis changed during follow-up. A flow diagram of the enrolled patients is shown in Fig. 1.

### Definitions and study procedure

All enrolled patients were divided into two subgroups based on the criteria: patients with at least a 20% increase in the initial maximal diameter of the tumors during follow-up were included in the progressive disease group; the other patients were assigned to the stable disease group. We compared patient demographics, initial tumor size, tumor location, ultrasonographic features, and growth rate between the two groups at 1 year, 2 years and more than 3 years respectively. In the receiver operating characteristic (ROC) curve analysis, the progressive disease cases were defined as the true positives, and the stable disease cases were defined as the true negatives when determining the cut-off value of the optimal initial size for medical intervention.

### Statistical analysis

All statistical analyses were performed with SPSS statistical software (version 20.0 for Windows; SPSS, Chicago, IL, USA). Numerical variables were expressed as the mean ± SD unless otherwise stated. Discrete variables were analyzed using the Chi-square test or Fisher’s exact test. The optimal cut-off values for tumor size as a prognostic variable were chosen from a ROC curve with the criterion variable “tumor size” and “progressive disease” as condition variables. We considered *P* values <0.05 to be statistically significant for a two-sided test.

## Results

### Patient characteristics

A total of 74 patients were diagnosed with minimal gastric GISTs by using EUS meeting the included criteria; 5 patients were excluded for diagnoses changed during the follow-up. Finally, 69 patients met the criteria for enrolment (see Table 1). The average age was 59 (range, 27–84) years. There were 17 (21.8%) men and 52 (66.7%) women. Tumors were located at the cardia in 7 patients (9.0%), at the fundus in 43 (55.1%) patients, at the body in 18 (23.1%) patients, and at the pylori in 1 (1.3%) patient. The mean initial tumor size was 8.8 (range, 3–20) mm. Only 4 cases’ (5.8%) initial EUS features have the high risk feature, such as heterogeneous echo texture, irregular extraluminal border, echogenic foci, and anechoic space. 5 cases (7.2%) were identified by successfully performing pathological examination of EUS-FNA. The mean EUS follow-up period was 28 months (range 12–70 months). Of the 69 EUS-suspected GISTs, 16 (23.2%) showed significant changes in size (see Table 2). The tumors were mainly located in the gastric body (9 cases, 56.3%) and fundus (7 cases, 43.7%). Among the cases, 11 patients underwent resection, and all their tumors proved to be GISTs. While the other 5 patients refused surgery and were followed up. Out of 11 patients, 4 patients had lesions with higher malignant potential, reflected by mitotic rates of more than 5 per 50 high-power fields (HPFs). Molecular analysis revealed KIT exon 11 mutation in 10 cases, and wild type in 1 cases. Moreover, of the 69 EUS-suspected GISTs, significant change in echo patterns was observed in 8 patients (11.6%). 6 cases (75%) showed significant changes in size.

**Table 1:** Clinical characteristics of patients

Characteristics	Cases (*n* = 69)
Age (years)	58 (59, 27–84)^a^
Gender, *n* (%)	
Male	17 (21.8%)
Female	52 (66.7%)
Tumor location	
Cardia	7 (9.0%)
Fundus	43 (55.1%)
Body	18 (23.1%)
Antrum	1 (1.3%)
Initial diameter (mm)	8 (8.8, 3–20)^a^
Initial EUS features, *n* (%)	
Low-risk features	65 (94.2%)
High-risk features	4 (5.8%)
Follow-up duration (mo)	23 (28, 12–70)^a^
≥ 12 months, *n* (%)	69 (100%)
≥ 24 months, *n* (%)	43 (62.3%)
≥ 36 months, *n* (%)	30 (43.5%)

^a^Median (mean, range)

**Table 2:** Characteristics of the GISTs that changed in size

No.	Sex	Age	Site	Initial Size	F/u Size	Initial EUS	F/u EUS	Treatment	Final Diagnosis	Mitotic count	Mutation type
1	M	72	Fundus	6	17	Low risk	Low risk	OP	GIST	7/50HPF	KIT exon 11
2	F	75	Body	16	30	Low risk	High risk	OP	GIST	6/50HPF	KIT exon 11
3	M	71	Body	20	40	Low risk	High risk	OP	GIST	10/50HPF	KIT exon 11
4	F	51	Fundus	8	15	Low risk	High risk	OP	GIST	0/50HPF	KIT exon 11
5	F	83	Fundus	7	15	Low risk	Low risk	Surveillance	Not available	Not available	Not available
6	F	78	Body	11	22	Low risk	High risk	OP	GIST	2/50HPF	KIT exon 11
7	M	67	Body	12	17	Low risk	Low risk	OP	GIST	3/50HPF	KIT exon 11
8	M	57	Fundus	10	12	Low risk	Low risk	Surveillance	Not available	Not available	Not available
9	F	51	Fundus	10	13	Low risk	Low risk	Surveillance	Not available	Not available	Not available
10	F	53	Body	11	15	Low risk	Low risk	Surveillance	Not available	Not available	Not available
11	M	84	Body	18	35	Low risk	High risk	OP	GIST	3/50HPF	KIT exon 11
12	F	57	Body	11	14	Low risk	Low risk	Surveillance	Not available	Not available	Not available
13	M	67	Body	15	30	Low risk	Low risk	OP	GIST	6/50HPF	KIT exon 11
14	M	65	Fundus	10	24	Low risk	High risk	OP	GIST	3/50HPF	KIT exon 11
15	F	59	Body	12	15	Low risk	Low risk	OP	GIST	0/50HPF	KIT exon 11
16	F	51	Fundus	8	13	High risk	High risk	OP	GIST	2/50HPF	Wild-type

*Abbreviations*: *F/u* indicates follow-up, *HR* high risk, *IR* intermediate risk, *LR* low risk, *OP* operation

### Analysis of the two groups

Out of all, 69, 43 and 30 patients had been followed up more than 1 year, 2 years and 3 years respectively. When all the patients were followed up to 1 years, according to the criteria, there were 58 (84.1%) patients in the stable disease group and 9 (15.9%) patients in the progressive disease group (see Table 3). Both groups were similar in gender, tumor location, and initial EUS features. The mean age (67.9 vs. 57.6, *p* = 0.012), initial diameter (12.6 mm vs. 8.1 mm, *p* = 0.000), and follow-up EUS high risk features (45.5% vs. 8.6%, *p* = 0.001) significantly predicted progressive disease compared with the stable disease group. The mean value for the average tumor growth rate per annum in the progressive disease group was 50.7%, which was significantly higher than the −1.2% rate in the stable disease group (*p* = 0.000). The data for the two groups are presented in Table 1. When the patients were followed up to 2 years and more than 3 years (see Tables 4 and 5), we could find similar results that the age, initial diameter and follow-up EUS high risk features were significantly predicted progressive disease.

**Table 3:** Characteristics of the minimal EUS-suspected GISTs followed up to 1 year

Characterisitics	Stable disease group (*n* = 58)	Progress disease group (*n* = 11)	*P*
Age	57.6 ± 12.3	67.9 ± 11.2	0.012
Gender, *n* (%)			0.053
Male	9 (17.6%)	8 (44.4%)	
Female	42 (82.4%)	10 (55.6%)	
Tumor location, *n* (%)			
Cardia	7 (12.1%)	0	0.587
Fundus	39 (67.2%)	4 (36.4%)	0.087
Body	11 (19.0%)	7 (63.6%)	0.005
Antrum	1 (1.7%)	0	1.000
Initial diameter (mm)	8.07 ± 3.11	12.55 ± 4.16	0.000
Initial EUS, *n* (%)			0.509
Low risk	55 (94.8%)	10 (90.9%)	
High risk	3 (5.2%)	1 (9.1%)	
F/u EUS, *n* (%)			0.001
Low risk	53 (91.4%)	6 (54.5%)	
High risk	5 (8.6%)	5 (45.5%)	
Growth rate per year (%)	−1.2 ± 9.7	50.7 ± 33.6	0.000

**Table 4:** Characteristics of the minimal EUS-suspected GISTs followed up to 2 years

Characterisitics	Stable disease group (*n* = 37)	Progress disease group (*n* = 6)	*P*
Age	58.0 ± 11.8	70.7 ± 11.6	0.019
Gender, *n* (%)			0.164
Male	8 (17.6%)	3 (44.4%)	
Female	29 (82.4%)	3 (55.6%)	
Tumor location, *n* (%)			
Cardia	6 (16.2%)	0	0.571
Fundus	25 (67.6%)	2 (33.3%)	0.174
Body	6 (16.2%)	4 (66.7%)	0.020
Antrum	0	0	1.000
Initial diameter (mm)	8.14 ± 3.10	13.83 ± 4.83	0.033
Initial EUS, *n* (%)			1.000
Low risk	35 (94.6%)	6 (100%)	
High risk	2 (5.4%)	0	
F/u EUS, *n* (%)			0.007
Low risk	33 (89.2%)	2 (33.3%)	
High risk	4 (10.8%)	4 (66.7%)	
Growth rate per year (%)	−0.8 ± 5.4	37.3 ± 22.1	0.008

**Table 5:** Characteristics of the minimal EUS-suspected GISTs followed up to more than 3 years

Characterisitics	Stable disease group (*n* = 23)	Progress disease group (*n* = 7)	*P*
Age	59.4 ± 11.8	62.9 ± 10.7	0.487
Gender, *n* (%)			0.345
Male	5 (21.7%)	3 (42.9%)	
Female	18 (78.3%)	4 (57.1%)	
Tumor location, *n* (%)			
Cardia	4 (17.4%)	0	0.548
Fundus	16 (69.6%)	4 (57.1%)	0.657
Body	3 (13.0%)	3 (42.9%)	0.120
Antrum	0	0	1.000
Initial diameter (mm)	7.70 ± 2.79	11.86 ± 4.78	0.007
Initial EUS, *n* (%)			1.000
Low risk	22 (95.7%)	7 (100%)	
High risk	1 (4.3%)	0	
F/u EUS, *n* (%)			0.031
Low risk	22 (95.7%)	4 (57.1%)	
High risk	1 (4.3%)	3 (42.9%)	
Growth rate per year (%)	−0.5 ± 3.1	19.1 ± 9.1	0.001

### ROC curve analysis

**Fig. 2:**
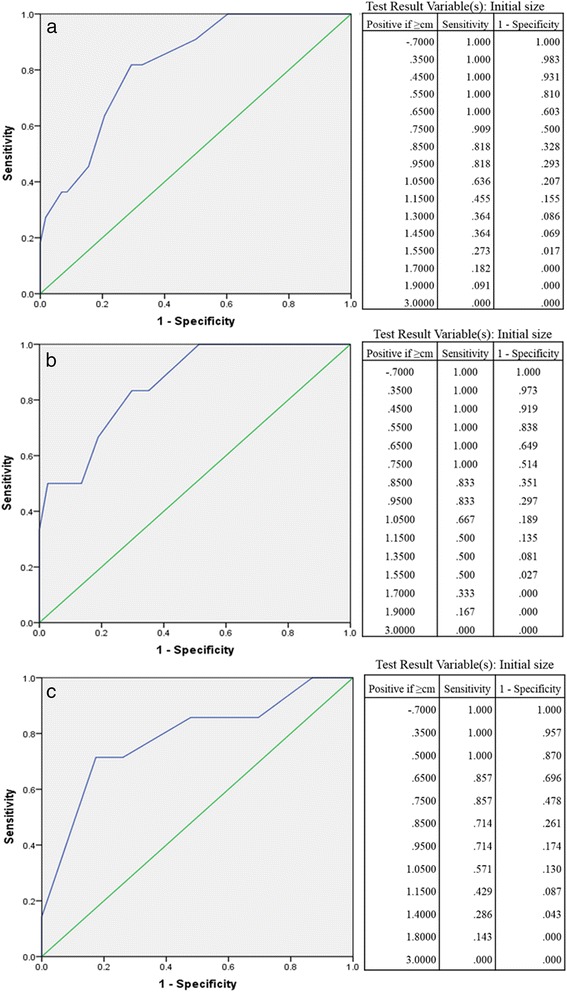
Receiver operating characteristic curve. All the receiver operating characteristic curve analyses showed that the tumor size cut-off was 9.5 mm, at 1 year (**a**), 2 years (**b**), and more than 3 years (**c**)

We generated ROC curves to find best the sensitivity and specificity to detect the optimal cut-off value for predicting potential tumor growth. For 1 year follow-up, The area under the curve (AUC) was 0.818, indicating that the best cut-off value of tumor size was 9.5 mm. The sensitivity, specificity, positive predictive value, negative predictive value, and consistency rates were 81.8, 70.7, 34.6, 95.3, 72.5%, respectively (see Fig. 2a). For 2 years and more than 3 years follow-up, the best cut-off value of tumor size was also 9.5 mm. The AUC, sensitivity, specificity, positive predictive value, negative predictive value, and consistency rates were 0.858, 83.3, 70.3, 31.3, 96.3, 69.8 and 0.786, 82.6, 73.3, 55.6, 90.5, 80.0%, respectively.(see Fig. 2b, c).

### Surveillance timing

Eleven out of 69 GISTs (15.9%), 6 out of 43 GISTs (14.0%), 7 out of 30 GISTs (23.3%) showed significant changes in size, at 1 year, 2 years, and more than 3 years respectively. With the 9.5 mm cut-off, there was a significant difference between the two subgroups. Only 4.7% (2/43) and 3.7% (1/27) of gastric EUS-suspected GISTs of <9.5 mm in size showed significant changes at 1 year and 2 years, while 9.5% (2/21) at more than 3 years. 34.6% (9/26), 31.3% (5/16) and 55.6% (5/9) of gastric EUS-suspected GISTs of ≥ 9.5 mm in size showed significant changes at 1 year, 2 years and more than 3 years.

## Discussion

For a long time, the actual incidence of gastrointestinal stromal tumor (GIST) was underestimated. With the improvements in understanding of this disease and examination methods, the detection rate was increasing. Most recent studies have suggested the incidence of gastric GISTs to be between 10 and 20 cases per million, which is 2–3 times more than the data of 20 years ago [4].This trend was also showed in minimal GIST especially for gastric GIST. Based on the data 20 years ago from Armed Forces Institute of Pathology (AFIP) of US, it has been reported that of the 1687 tumors with size measurements, 127 (7.5%) were 2 cm or smaller [5]. While the data 10 years later from the REGISTER study of Italy showed the rate was up to 18.3%(170/929) [6]. Moreover, A study from Germany showed among the consecutive 98 autopsy cases, miro-GIST (less than 1 cm) were found in 22 patients (22.5%) [7]. A latest population-Based study [8] for milli-GIST (<2 cm) in the National Cancer Institute’s SEER database showed that the annual incidence rate of gastric milli-GISTs was 2.6 per 10 million.

Because the natural course of minimal gastric GIST remains largely unknown, the current management policy for gastric GISTs <2 cm is usually conservative, unless tumors grow more than 2 cm or symptoms occur such as bleeding, acute abdomen, etc. [9]. Despite the patients with minimal gastric GIST are recommend for close surveillance, there are some difficulties in clinical works. Firstly, some patients would feel anxiety, depression and stress for survival with tumor. Secondly, some patients could not be followed up regularly for their poor compliance. These cause some patients to choose the two extremes: excision by overtreatment or few surveillance with delayed treatment. Therefore, identification of malignant potential for minimal gastric GIST is very important. Although most milli-GISTs are presumed to have less malignant potential especially which with low mitotic rate. If with high mitotic rate, the metastases rate or tumor-related mortality would be significantly worse. EUS-guided fine needle aspiration (EUS-FNA) has been suggested for the determination of malignant potential in gastric GISTs with high accuracy (91.7–97%) [10, 11]. While in these study, the GISTs evaluated were larger ones >2 cm mostly (80–91%). It would have been difficult to use EUS-FNA for minimal gastric GISTs. Due to the small size, success ratio of puncture maybe low. Moreover there would be insufficient tissues obtained by biopsy to assess mitotic or genetic mutation. Mekky et al. reported adequate samples were obtained in 67.6% of gastric submucosal tumors with size <20 mm [12]. Even the adequate specimens were considered, the reported diagnostic yields for tumors less than 20 mm was 71% [11]. Thus, the diagnosis of miminal GISTs may be mostly diagnosed based on EUS appearance. Ultrasonographic features are another important predictive factor of malignancy. High-risk EUS features include irregular border, cystic spaces, ulceration, echogenic foci, and heterogeneity [13]. However, ultrasonographic features might not have been in smaller lesions as sensitive as for larger ones. In our study, there was no difference between stable disease group and progressive disease group regarding initial malignant ultrasonographic features. While we found 8 cases showed ultrasonographic feature changes. They all occurred after the diameter increased more than 1 cm. Among them 6 (75.0%) showed significant changes in size.

With knowledge of GIST biological behaviours, we gradually realized that the malignancy potential of some minimal gastric GISTs was high, and these need to have medical intervention. Even some experts suggested surgical resection of all minimal gastric GISTs once diagnosed [14]. The growth of the tumor is an important index of malignant potential. There are some studies proposing a cut-off value of initial tumor size, in order to predicting GISTs with significant-sized change. A retrospective analysis by Lachter et al. [15] reported that out of 70 GISTs monitored by EUS, enlargement in size was detected significantly more in GISTs over 17 mm diameter (*P* < 0.018) at averaged follow-up examination 23.2 mm. In another retrospective study by Gill et al. [16], the majority (86.3%) of <3 cm upper gastrointestinal subepithelial tumors (SETs) did not increase in size and/or change in echogenic features during a median of 23 months. In another study by Kim et al. [17], in 989 gastric subepithelial tumors, SETs of 10 to 30 mm in size grew significantly more rapidly than SETs <10 mm over a median period of 24 months when followed up endoscopically or by EUS. Fang et al. [18] followed 50 patients with EUS-suspected gastric GISTs of sizes less than 3 cm over a period of more than 24 months (range 24–101 months), found that the best cutoff size associated with tumor progression was 1.4 cm having an 85.7% sensitivity, 86.1% specificity, and 86.0% accuracy. While all these studies included the GISTs or SETs >2 cm. These tumors should be resected without controversy. The current surveillance and management policy for gastric minimal GISTs is still controversial. So our study was specific for GISTs ≤2 cm of the stomach. Moreover most studies above followed the patients over much different time (range 3 months to more than 5 years). This may increase select bias, because the possibility of significant increase in tumor size is different over different time. In this study, all the patients were followed up over the same time (1 year, 2 years or more than 3 years). It was also helpful to propose appropriate timing for endoscopic ultrasonography (EUS) follow-up. We found Minimal EUS-suspected GISTs larger than 9.5 mm may be associated with significant progression.

Experts recommend EUS surveillance of gastric milli-GISTs, although there are few data to support surveillance at all. In 2015, NCCN recommended for the patients with gastric minimal GISTs, EUS surveillance at 6–12 moths intervals may be considered [19]. The study cited by NCCN analyzed the data of 37 patients with GISTs, while only 13.5% (5/37) of all GISTs showed <2 cm in size. Due to insufficient evidences, the latest edition of the guide by NCCN [9] modified its recommendation description to “consider periodic endoscopic surveillance”. This new study cited by NCCN was designed as an online survey from all 413 members of the American Society for Gastrointestinal Endoscopy (ASGE) EUS Special Interest Group. It showed for lesions not resected, 70% survey annually, 19% less than annually, 10% more than annually, and 1% do not survey [20]. While this studies also included a lot of GISTs >2 cm. Based on experts’ preferences, ASGE recommended annual surveillance is commonly practiced [21]. In our study, 15.9, 14.0 and 23.3% GISTs showed significant changes in size, at 1 year, 2 years, and more than 3 years respectively. So we also recommend endoscopic surveillance annually for all gastric milli-GISTs. In addition, with the 9.5 mm cut-off, few of GISTs <9.5 mm (<5%) showed significant size changes in the first 2 years. On the contrary, numerous GISTs ≥ 9.5 mm showed significant changes even in the first year. So ≥ 9.5 mm GIST maybe need a EUS less than annually. For the patients with <9.5 mm GIST, EUS surveillance at 2–3 years interval may be considered. This strategy may increase the quality of life and enhance the compliance for patients with smaller milli-GIST.

Some limitations existed in our study. First, few cases (5/69) enrolled in the study were identified by successfully performing pathological examination of EUS-FNA, due to technical difficulty or insufficient material to make diagnosis. Although the accuracy of GIST diagnosed by using EUS is as high as 87%, [2, 3] this would influence the results of our study because of some them might have had benign submucosal tumors, such as leiomyoma. While in our data, out of 16 tumors with significant increase in tumor size located in the fourth layer on EUS, 11 underwent surgical resection and final pathological diagnosis was all GISTs. Second, this is a single-institution retrospective study. Further confirmation of these findings is needed in a larger, multicentre cohort.

## Conclusions

In conclusion, an initial tumor size larger than 9.5 mm was associated with significant tumor progression and was valuable for predicting the malignant potential GIST. According to our results, the patients with a <9.5 mm GIST may have a EUS extended to every 2–3 years, while ≥ 9.5 mm GIST should have a EUS 6–12 months.

## Additional files

Additional file 1: Characteristics of all the GISTs. This supplementary file includes clinical parameters of all patients. (XLSX 20 kb)
Additional file 2: Clinicopathological features of all the PD GISTs. It contains data on clinicopathological features of patients with progressive disease (PD). (XLSX 11 kb)

## Abbreviations

AFIP: Armed Forces Institute of Pathology
ASGE: American Society for Gastrointestinal Endoscopy
AUC: Area under the curve
ESMO: European Society of Medical Oncology
EUS: Endoscopic ultrasonography
FNA: Fine needle aspiration
GIST: Gastrointestinal stromal tumor
HADS: Hospital anxiety depression scale
NCCN: National Comprehensive Cancer Network
ROC: Receiver operating characteristic
SET: Subepithelial tumor
